# 1178. Opportunities and Challenges to Emergency Department-Based HIV Testing Services and Self-Testing Programs: A Qualitative Study of Healthcare Providers and Patients in Kenya

**DOI:** 10.1093/ofid/ofac492.1015

**Published:** 2022-12-15

**Authors:** Adam Aluisio, Scarlett Bergam, Agot Ogory, Beatrice Ngila, Janet Sugut, Rose Bosire, David Bukusi, John Kinuthia, Kate Guthrie, David Katz, Carey Farquhar, Michael Mello

**Affiliations:** Alpert Medical School of Brown University, Providence, Rhode Island; George Washington University School of Medicine and Health Sciences, Washington, District of Columbia; Kenyatta National Hospital, Nairobi, Nairobi Area, Kenya; Kenyatta National Hospital, Nairobi, Nairobi Area, Kenya; Kenyatta National Hospital, Nairobi, Nairobi Area, Kenya; Kenya Medical Research Institute, Nairobi, Nairobi Area, Kenya; Kenya Medical Research Institute, Nairobi, Nairobi Area, Kenya; Kenyatta National Hospital, Nairobi, Nairobi Area, Kenya; Alpert Medical School, Brown University, Providence, Rhode Island; University of Washington, Seattle, Washington; University of Washington, Seattle, Washington; Alpert Medical School of Brown University, Providence, Rhode Island

## Abstract

**Background:**

Young people in Sub-Saharan Africa, especially males, have been insufficiently engaged through HIV Testing Services (HTS). In Kenya, younger persons are often treated in emergency departments (EDs) for injuries, an interaction where HTS and HIV self-testing (HIVST) can be leveraged. Data from stakeholders on ED-HTS and HIVST is lacking and needed to understand opportunities and barriers for HIV testing and care, and inform program implementation.

**Methods:**

Between December 2021 and March 2022, 32 in-depth interviews (IDIs) were conducted with 16 male and 16 female patients who had been treated in the Kenyatta National Hospital (KNH) ED, half of whom had been HIV-tested. Six focus-group discussions (FGDs) were also conducted with 50 nurses, doctors, HIV testing counselors, and administrators working in the ED. All transcripts were double-coded and thematically analyzed using Dedoose software and a parallel inductive and deductive coding approach which allowed for capture of both *a priori* and emergent themes.

**Results:**

Patients and providers agreed that ED-HTS are facilitated by friendly staff, patient education, high perceived HIV risk, and confidentiality. However, ED-HTS is limited by burdens on staff, resources, time, and space, as well as severity of patient injuries limiting ability to consent to or prioritize HIV testing. These limitations provide opportunities for ED-HIVST: particularly the ability to test at a comfortable time and place, especially when provided alongside sufficient HIV and testing education, contact with healthcare providers, and psychosocial support. Barriers for ED-HIVST where identified and as patients’ concerns about HIVST accuracy and mental health impacts of a positive test, as well providers’ identified barriers on their concerns for loss to follow up and inability to complete confirmatory testing.

COM-B Model

**Figure:**
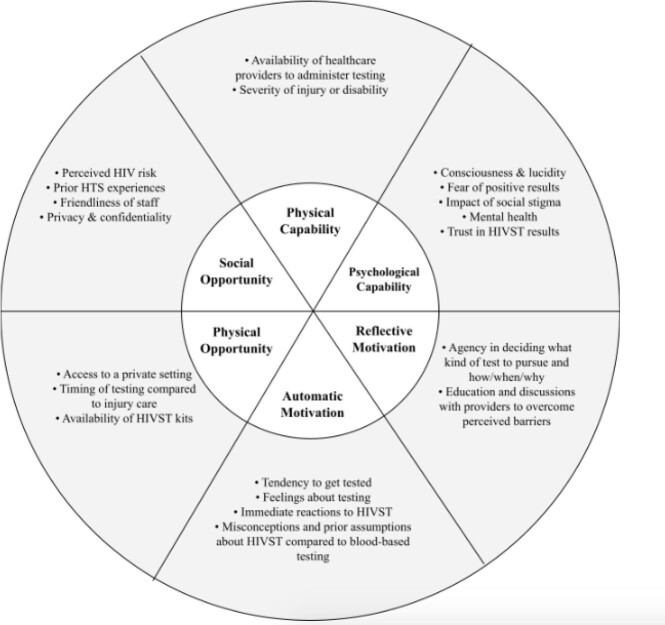

Application of the COM-B Model of Behavior Change to ED-HIVST Acceptability in Kenya

**Conclusion:**

ED stakeholders are receptive to HTS and HIVST, and patients desire the opportunity to use HIVST. Potential challenges—such as psychological effects of testing positive, worries about access to follow-up care, and confusion about how to self-administer testing, may be addressed through programming designed to promote education, access and ensure follow-up mechanisms.

**Disclosures:**

**All Authors**: No reported disclosures.

